# RhoA/ROCK-dependent moesin phosphorylation regulates AGE-induced endothelial cellular response

**DOI:** 10.1186/1475-2840-11-7

**Published:** 2012-01-17

**Authors:** Jiping Wang, Hongxia Liu, Bo Chen, Qiang Li, Xuliang Huang, Liqun Wang, Xiaohua Guo, Qiaobing Huang

**Affiliations:** 1 Department of Pathophysiology, Key Lab for Shock and Microcirculation Research, Southern Medical University, Guangzhou, 510515, P. R. China

**Keywords:** advanced glycation end products (AGEs), vascular permeability, RhoA/ROCK pathway, moesin

## Abstract

**Background:**

The role of advanced glycation end products (AGEs) in the development of diabetes, especially diabetic complications, has been emphasized in many reports. Accumulation of AGEs in the vasculature triggers a series of morphological and functional changes in endothelial cells (ECs) and induces an increase of endothelial permeability. This study was to investigate the involvement of RhoA/ROCK-dependent moesin phosphorylation in endothelial abnormalities induced by AGEs.

**Methods:**

Using human dermal microvascular endothelial cells (HMVECs), the effects of human serum albumin modified-AGEs (AGE-HSA) on the endothelium were assessed by measuring monolayer permeability and staining of F-actin in HMVECs. Activations of RhoA and ROCK were determined by a luminescence-based assay and immunoblotting. Transfection of recombinant adenovirus that was dominant negative for RhoA (RhoA N19) was done to down-regulate RhoA expression, while adenovirus with constitutively activated RhoA (RhoA L63) was transfected to cause overexpression of RhoA in HMVECs. H-1152 was employed to specifically block activation of ROCK. Co-immunoprecipitation was used to further confirm the interaction of ROCK and its downstream target moesin. To identify AGE/ROCK-induced phosphorylation site in moesin, two mutants pcDNA3/HA-moesinT^558A ^and pcDNA3/HA-moesinT^558D ^were applied in endothelial cells.

**Results:**

The results showed that AGE-HSA increased the permeability of HMVEC monolayer and triggered the formation of F-actin-positive stress fibers. AGE-HSA enhanced RhoA activity as well as phosphorylation of ROCK in a time- and dose-dependent manner. Down-regulation of RhoA expression with RhoA N19 transfection abolished these AGE-induced changes, while transfection of RhoA L63 reproduced the AGE-evoked changes. H-1152 attenuated the AGE-induced alteration in monolayer permeability and cytoskeleton. The results also confirmed the AGE-induced direct interaction of ROCK and moesin. Thr558 was further identified as the phosphorylating site of moesin in AGE-evoked endothelial responses.

**Conclusion:**

These results confirm the involvement of RhoA/ROCK pathway and subsequent moesin Thr558 phosphorylation in AGE-mediated endothelial dysfunction.

## Background

Advanced glycation end products (AGEs) are a heterogeneous group of complex compounds that are formed irreversibly in serum and tissues via a chain of non-enzymatic chemical reactions [1]. The role of AGEs in the development of diabetes, especially diabetic complications, has been emphasized in many reports [2,3] and the level of AGEs is correlated with the severity of diabetic complications [4-7]. Direct effects of AGEs include formation of extracellular cross-links that may trap various unrelated macromolecules. Furthermore, AGEs can bind to various receptors, such as the receptor for AGE (RAGE) or AGE receptors 1-3 (AGER1-3), leading to complex effects on cellular function via complicated transduction pathways [8-10]. The major AGE receptor (RAGE) enhances inflammation, while AGER1 promotes the removal of AGEs and blocks inflammation [11]. The vascular endothelium is the front-line organ for vascular injury and a common target of various risk factors, with dysfunction of the microvascular endothelial barrier playing a critical role in the pathogenesis of insulin resistance and diabetes [12]. Accumulation of AGEs in the vasculature triggers a series of morphological and functional changes in endothelial cells (ECs) and induces an increase of endothelial permeability [13]. It has been reported that AGEs cause significant disorganizations of the F-actin cytoskeleton, disruption of tight junctions and adherens junctions in cultured human umbilical venous endothelial cells (HUVECs), and increase the permeability of EC monolayers [14,15]. The Rho family of small GTPase proteins control a wide variety of cellular processes. RhoA is one of the best-known members of this family and the Rho kinases (ROCK) are the first and the best-characterized RhoA effectors. By modulating the organization of the actin cytoskeleton, RhoA/ROCK signaling regulates a wide range of cellular functions, such as contraction, motility, proliferation, and apoptosis. It has been shown that ROCK-dependent re-arrangement of the actin cytoskeleton and changes of cell contractility are involved in the regulation of endothelial permeability [16-19]. Our previous studies have also suggested the involvement of ROCK in AGE-induced endothelial responses. Inhibition of ROCK with Y-27632 was reported to reduce the AGE-evoked formation of actin stress fibers and the weakening of adherens junction [15] in HUVECs. Y-27632 also abolished the AGE-induced increase of ROCK phosphorylation [20]. Using anti-RAGE antibody, we and Hirose et al. have demonstrated in HMVECs and HUVECs, respectively, that activation of RhoA depends on the binding of AGEs to RAGE [14,20].

Since ROCK does not directly act on F-actin and other cytoskeletal molecules, there is a missing link between the activation of ROCK and the cytoskeleton reorganization. ERM (ezrin/radixin/moesin) proteins are emerging as the potential candidates that likely mediate this process. Serving as cross-linkers between actin filaments and plasma membrane, ERM molecules are engaged in cell adhesion, microvilli formation, cell motility, etc [21-23]. Moesin is regarded as the most important ERM in endothelia since it is the dominant ERM expressed in endothelial cells [8,24]. It has been demonstrated by several experiments that Rho-ROCK is a typical upstream pathway for the phosphorylation of moesin [23]. On the basis of in vitro and in vivo studies [20,25], Rho kinase is also postulated to phosphorylate moesin in AGE-induced endothelial response, but there is still a lack of direct evidence for the interaction of ROCK and moesin in AGE-evoked endothelial alteration.

One of the purposes of this study was to further investigate the specific effect of RhoA/ROCK pathway on endothelial responses to AGEs. By transfection of recombinant adenoviruses targeting RhoA activity and usage of a more specific ROCK inhibitor, we explored the contribution of this signaling pathway to the AGE-induced increase of endothelial monolayer permeability and alterations of the F-actin cytoskeleton by clarifying the phosphorylation state of RhoA and ROCK after exposure to AGEs. This present study is also aimed to further confirm the interaction of ROCK and its downstream target moesin by using Co-immunoprecipitation (Co-IP). To identify the AGE/ROCK-induced phosphorylation site in moesin, two mutants pcDNA3/HA-moesinT^558A ^and pcDNA3/HA-moesinT^558D ^were applied in endothelial cells and the AGE-stimulated responses were compared.

## Materials and methods

### Chemicals and reagents

A G-LISA™ RhoA activation assay kit was purchased from Cytoskeleton (Denver, USA). Antibody recognizing total ROCK, ROCK inhibitor H-1152, and protein G plus/protein A agarose suspension were obtained from Calbiochem (USA), while antibody recognizing p-ROCK came from Upstate (NY, USA). RhoA N19 recombinant adenovirus (dominant negative) and RhoA L63 recombinant adenovirus (constitutively active) were purchased from Cell Biolabs (San Diego, CA, USA). Antibody recognizing moesin was purchased from Abcam (Abcam, UK) while anti-phosphorylated (p-)moesin (Thr558) antibody was purchased from Santa Cruz Biotechnology (Santa Cruz, CA). Antibody recognizing HA was purchased from Cell Signaling Technology (CST, USA). Lipofectamine™ LTX and PLUS™ reagents were obtained from Invitrogen Technology. Primers were designed using Primer Premier 5 software and synthesized by Invitrogen (USA). Restriction enzymes EcoR I and Xho I were from Takara (Japan). High Fidelity DNA polymerase KOD-Plus, KOD-Plus-Mutagenesis kit and Thunderbird SYBR qPCR Mix were obtained from Toyobo (Japan). Rhodamine-phalloidin was obtained from Molecular Probe (Carlsbad, CA, USA). MCDB 191 medium, DMEM medium, fetal bovine serum (FBS), trypsin, glutamine, penicillin, and streptomycin were all from Gibco BRL (Grand Island, NY, USA). Chemicals were purchased from Sigma (St. Louis, MO, USA) unless otherwise indicated.

### Preparation of AGE-HSA

Advanced glycation end product-modified human serum albumin (AGE-HSA) was prepared as previously reported [20], essentially according to the protocol of Hou et al [26,27]. Briefly, human serum albumin (150 mmol/L, pH 7.4) was incubated in PBS with D-glucose (250 mmol/L) at 37°C for 8 weeks, while control albumin was incubated without glucose. After the incubation period, both solutions were extensively dialyzed against PBS and purified. The endotoxin content was measured with a limulus amebocyte lysate assay (Sigma, St. Louis, MO, USA) and was found to be less than 500 U/L in both solutions. AGE-specific fluorescence was determined by ratio spectrofluorometry, showing that AGE-HSA had an AGE content of 74.802 U/mg protein, while native albumin had an AGE content of less than 0.9 U/mg protein.

### Cells and culture conditions

A human dermal microvascular endothelial cell (HMVEC) line was purchased from Cell Applications (San Diego, CA) [28]. Cells were grown in 100-mm dishes or 6-well plates and were maintained in MCDB 131 containing endothelial cell growth supplements, 20% FBS, and 2 mmol/L L-glutamine at 37°C in a humidified atmosphere with 5% CO_2_. Primary human umbilical endothelial cells (HUVECs) (from Sciencell) were maintained in DMEM containing 10% FBS at 37°C in a humidified atmosphere with 5% CO_2._

### Stimulations of HMVECs

In all experiments, HMVECs were grown to 90% confluence and starved of serum for 2 hours before being stimulated with AGE-HSA at the indicated doses and for the indicated times. The AGE-HSA concentrations used in this experiment were based on data from our previous studies of HMVECs or freshly cultured primary HUVECs [15,20]. To clarify the involvement of RhoA in AGE-induced endothelial responses, HMVECs were infected with RhoA N19 recombinant adenovirus (dominant negative) according to the manufacturer's instructions at 24 h before stimulation with AGE-HSA. As the positive control, HMVECs were infected with RhoA L63 recombinant adenovirus (constitutively active) for 24 h before being studied. For inhibitor treatment, HMVECs were pretreated with H-1152 (20 μmol/L) and then cultured in fresh complete medium with 50 mg/L AGE-HSA for 8 h.

### Measurement of RhoA activity

RhoA activity was determined by using a luminescence-based G-LISA™ RhoA activation assay kit (Kit #BK121, Cytoskeleton, Inc., Denver, CO) according to the manufacturer's instructions. This assay employs a Rho-GTP-binding protein coating the wells of a 96-well plate. Active, GTP-bound Rho in cell lysates binds to the wells, while inactive GDP-bound Rho is removed through the washing steps. Then the bound active RhoA is detected by incubation with a specific RhoA antibody followed by an HRP-conjugated secondary antibody and a detection reagent, after which the luminescence is read on a microplate luminescence reader (MDC SpectraMax M5, USA). HMVECs were cultured in 6-well plates and treated as indicated above. Proteins were harvested by incubating the provided **c**ell lysis buffer with protease inhibitors, and centrifugating at 14,000 rpm at 4°C for 2 min to remove cell debris. The protein concentration was determined according to the manufacturer's protocol, and cell extracts were equalized to a protein concentration of 1.5 mg/ml for assay. Luminescence was detected according to the manufacturer's recommendations with minor modifications after incubation overnight at 4°C with the primary anti-RhoA antibody.

### ROCK and moesin phosphorylation and immunoblotting

Total cellular extracts were prepared by lysis and sonication of the cells in lysis buffer (20 mmol/L Tris pH 7.4, 2.5 mmol/L EDTA, 1% Triton X-100, 1% deoxycholic acid, 0.1% SDS, 100 mmol/L NaCl, 10 mmol/L NaF, 1 mmol/L Na_3_VO_4_) with protease and phosphatase inhibitors. Samples were subjected to SDS-PAGE, and proteins were transferred to polyvinylidene fluoride (PVDF) membranes. Blots were blocked with 5% bovine serum albumin in TBS containing 0.5% Tween 20 (TBS-T) for 1 h and then incubated with an 1:1000 dilution of primary antibody for p-ROCK (Upstate, NY, USA) or p-moesin (Santa Cruz, CA) overnight at 4°C on a rocker. After three washes for 5 min each with TBS-T, the blots were incubated with an 1:1000 dilution of HRP-conjugated species-specific respective secondary antibody for 1 h at room temperature. After washing three times for 5 min each with TBS-T, protein bands were visualized by chemiluminescence and then densitometric analysis was done by using Kodak IS2000R Imaging Station.

### Plasmid Constructs and Site-specific Mutagenesis

The full-length cDNA of moesin was obtained from HUVECs by RT-PCR and was ligated into eukaryotic expression vector pcDNA3/HA to obtain recombinant plasmid pcDNA3/HA-moesin, which was identified by EcoR I/Xho I double digestion and nucleotide sequencing. The point mutations of pcDNA3/HA-moesin were generated by inverse PCR with a site-specific mutagenesis kit (Toyobo, Japan) [29,30]. The two mutants, pcDNA3/HA-moesin^T558A ^and pcDNA3/HA-moesin^T558D^, were generated using primer A forward, 5' GCCAAGCAGCGCATTGACGAATTTGAGTC 3', and reverse, 5' GTTGCCCTGCCGGATCTGGCGC 3', and primer B forward, 5'GACAAGCAGCGCATTGACGAATTTGAGTCTATG 3', and reverse, 5' GTTGCCCTGCCGGATCTGGCGC 3', respectively. The mutations were identified by nucleotide sequencing.

### Transfection of plasmids to HUVECs

The transfection of plasmids was carried out by endofree plasmid midiprep kit. HUVECs of about 90%~95% confluent in 500 μl Opti-MEM medium without antibiotics were transfected with plasmids encoding HA-tagged two mutant forms of moesin (T558A and T558D) using Lipofectamine™ LTX and PLUS™ reagents (Invitrogen) according to the manufacturer's instructions. Briefly, for a 6-well format, 1 μg DNA was incubated in 500 μl Opti-MEM (antibiotics-free) while 8 μl lipofectamine LTX and 2 μl plus reagent were added and left at room temperature for 30 min. The cultured cells were washed once with Opti-MEM. The DNA-lipid complexes were added to the plates and incubated for 24 h, followed by stimulation with AGE-HSA (50 mg/L, 1 h). The cells were then used for Realtime PCR, immunoblotting, endothelial monolayer permeability assay, or immunofluorescence, respectively.

### Quantitative Realtime Reverse Transcription PCR

Total RNA was isolated from HUVECs by total RNA extraction kit (Biomegia USA) according to the manufacturer's protocol. Realtime PCR amplification was performed with moesin-specific primers. Total RNA of 1 μg was used as templates for cDNA synthesis in the reverse transcriptase reaction. After an initial 60 s predenature and 15 s denaturation at 95°C, cDNA was amplified for 40 cycles and then annealed at 60°C for 60 s for moesin and GAPDH. Under optimized conditions there was a single melting curve and no primer-dimer formation. The copy number for each mRNA was determined using a standard curve generated with external standards of known copy number. All the primers were designed using Primer Premier 5 software (Table 1).

**Table 1:** Primer sequences and size of Realtime PCR products

Gene	Primer	Product size (bp)
moesin surrounding primer	5' TAAACTTCGCCAACTCCGC 3'	2114
		
	5' TCCACACTTGACTCCCATAGG 3'	

moesin clone primer	5' CTAGAATTCGCGCCCAAAACGATCAGTGTG 3'	1735
		
	5' GCGCTCGAGTTACATAGACTCAAATTCGTC 3'	

moesin realtime primer	5' GATGTGCGGAAGGAAAGC 3'	158
		
	5' GTCTCAGGCGGGCAGTAA 3'	

GAPDH	5' GCACCGTCAAGGCTGAGAAC 3'	138
		
	5' TGGTGAAGACGCCAGTGGA 3'	

### Co-immunoprecipitation

80%~90% confluent HUVECs in 100 mm culture plate were used for Co-immunoprecipitation (Co-IP) after stimulated with AGE-HSA (50 mg/L for 1 h). Cells were lysed in the lysis buffer and the lysates were centrifuged at 10,000 g for 10 min at 4°C. Supernatants were incubated overnight at 4°C with ROCK antibody, moesin antibody or p-ROCK antibody, respectively, and then incubated with Protein A/G PLUS-Agarose (Merck, Darmstadt, Germany) for 3 h at 4^○^C. The bead-bound proteins were eluted with Laemmli's sample buffer after washing three times with lysis buffer. The eluted proteins were analyzed by immunoblotting with specific antibodies.

### Endothelial monolayer permeability assay

Endothelial monolayer permeability was measured as described by Tinsley [31]. ECs were grown to confluence on 1% gelatin-coated transwell clear polyester membranes (Corning Costar, Acton, MA, USA), and were exposed to the indicated reagents before stimulation with AGE-HSA. Then a tracer protein (TRITC-albumin, 1 g/L) was added to the upper chamber for 45 min, samples were then collected from both the upper (luminal) and lower (abluminal) chambers for fluorometry. The albumin concentration was measured by using a HTS 7000 microplate reader (Perkin-Elmer, Yokohama, Japan) and a standard curve. The permeability coefficient for albumin (Pa) was calculated as follows: Pa = [A]/t·1/A·V/[L], where [A] is the abluminal albumin concentration, t is the time in seconds, A is the membrane area in cm^2^, V is the volume of the abluminal chamber, and [L] is the luminal albumin concentration.

### F-actin staining

ECs were plated on gelatin-coated glass-bottomed microwell plates (MatTek, MA, USA) and cultured until confluence. After appropriate treatment, the cells were fixed and permeabilized for 15 min at room temperature in PBS with 3.7% formaldehyde and 0.5% Triton X-100. After a thorough wash with PBS, cells were incubated with rhodamine-phalloidin (2,000 U/L) for 40 min at room temperature. Then the cells were washed three times with PBS, and the plate was mounted for observation and imaging with a Leica TCS SP2 laser confocal scanning microscope (Wetzlar, Germany).

### Statistical analysis

Data were normalized to control values and are reported as a percentage of the baseline values (mean ± SD) for at least three independent experiments. Results were analyzed by one-way ANOVA followed by post hoc comparison. The level of significance was set at P < 0.05.

## Results

### AGE-HSA increase HMVEC monolayer permeability and stress fiber formation

**Figure 1:**
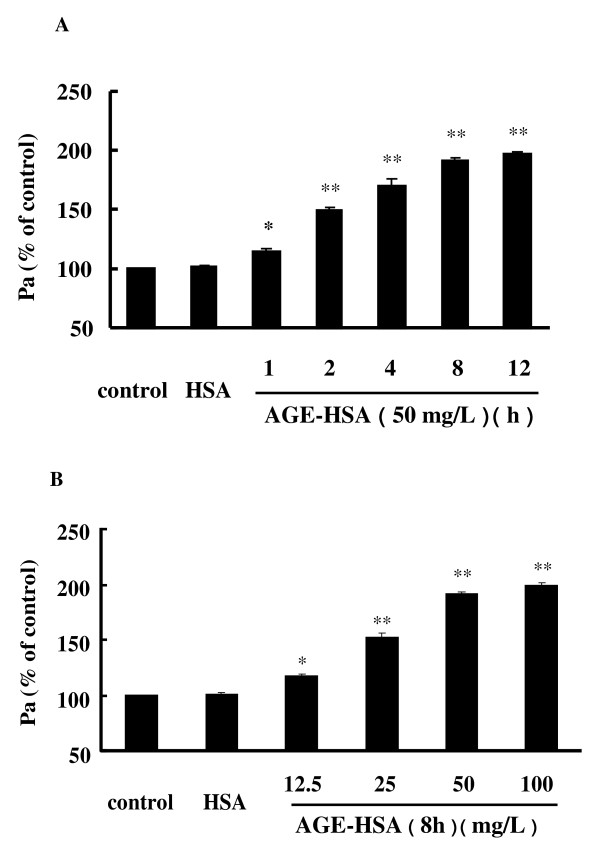
**AGE-HSA induced time-(A) and dose-(B) dependent increases of HMVEC monolayer permeability**. HMVECs were treated with 50 mg/L AGE-HSA for 1, 2, 4, 8, or 12 h, or with 12.5, 25, 50, or 100 mg/L AGE-HSA for 8 h. Culture medium was used as the blank control and HSA was used as the albumin control. Permeability was measured from the transflux of tracer protein TRITC-albumin across monolayers and was expressed as a coefficient for albumin (Pa). n = 3, *P < 0.05, **P < 0.01 *vs *control.

**Figure 2:**
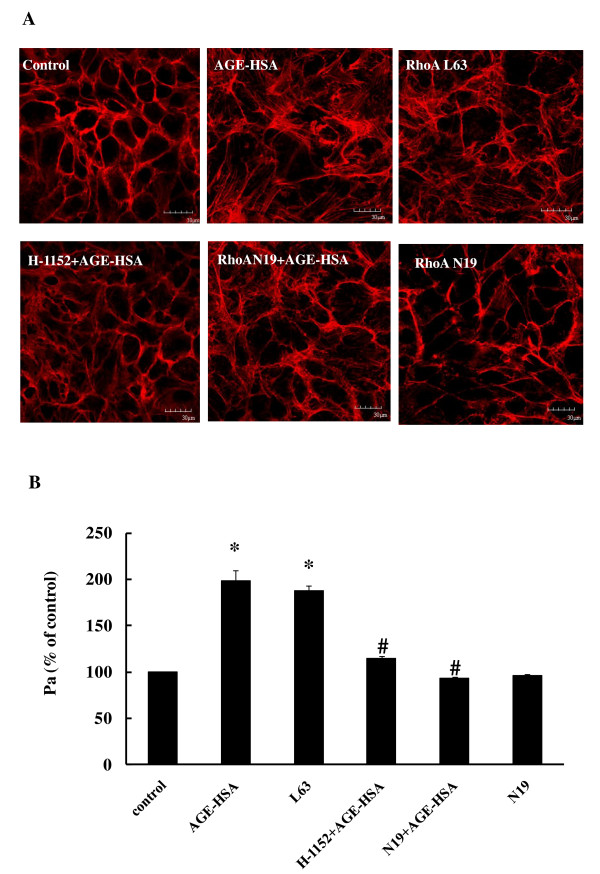
**Effect of down-regulation of the RhoA/ROCK pathway on AGE-induced F-actin redistribution (A) and increased monolayer permeability (B, *n = 3*)**. HMVECs was transfected with dominant negative human RhoA (RhoA N19) for 24 h with or without exposure to AGE-HSA (50 mg/L) for 8 h. In other groups, cells were transfected with either constitutively activated human RhoA (RhoA L63) for 24 h, or were treated with a ROCK inhibitor (H-1152) at 0.5 h before AGE-HSA addition. Culture medium was used as control. Representative confocal images of F-actin organization and formation of stress fibers are shown. Similar results were observed in 3 different cultures. *P < 0.05 *vs *control, #*P *< 0.05 *vs *AGE-HSA.

We have previously reported that AGE-HSA increase the permeability of monolayers and cause the formation of F-actin stress fiber in HUVECs [32]. This time, we confirmed the effects of AGEs on HMVECs by demonstrating that incubation with AGE-HSA led to hyperpermeability of HMVECs in a time- and dose-dependent fashion (Figure 1A and 1B). The permeability coefficient for albumin (Pa) was increased to 98.90 ± 10.47% after 8 h of exposure to AGE-HSA at 50 mg/L (*P *< 0.01 compared with the control) (Figure 2B). AGE-HSA also evoked the appearance of stress fibers in HMVECs (Figure 2A). In contrast, HSA alone did not cause any alterations of barrier function or the cytoskeleton in HMVECs.

### RhoA activation and ROCK phosphorylation induced by AGE-HSA

**Figure 3:**
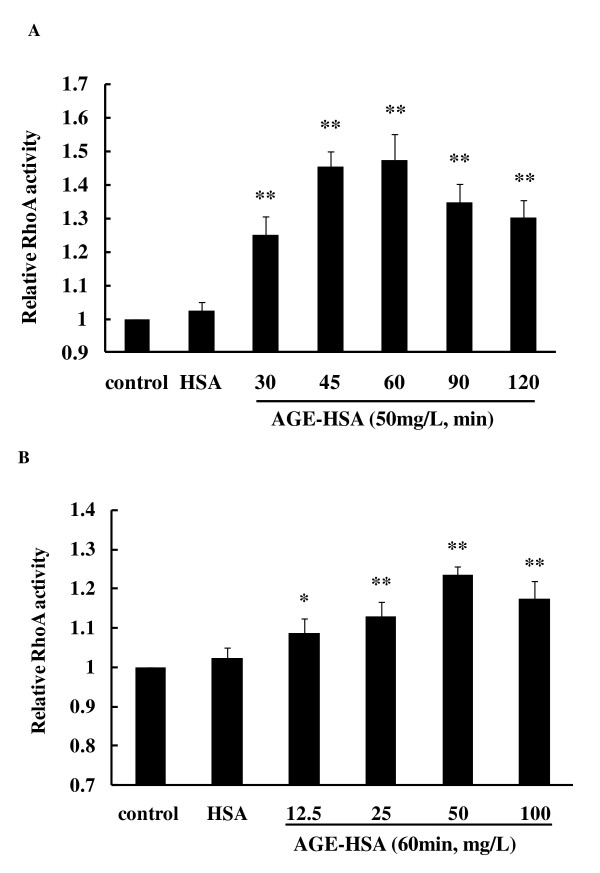
**AGE-HSA induced a time-(A) and dose-(B) dependent increase of RhoA activity**. HMVECs were treated with 50 mg/L AGE-HSA for 30, 45, 60, 90, or 120 min, or with 12.5, 25, 50, or 100 mg/L AGE-HSA for 120 min. RhoA activity was detected with a luminescence-based G-LISA™ assay. Activity reached its maximum at 60 min with 50 mg/L AGE-HSA. Culture medium was used as blank control and HSA was used as albumin control. *P < 0.05, **P < 0.01 *vs *control.

**Figure 4:**
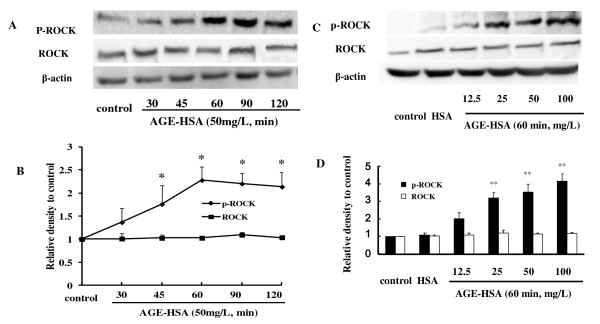
**AGE-HSA increased phosphorylation of ROCK in a time- and dose-dependent manner**. HMVECs were treated with 50 mg/L AGE-HSA for 30, 45, 60, 90, or 120 min (A), or with 12.5, 25, 50, or 100 mg/L AGE-HSA for 120 min (B). Phosphorylation of ROCK was detected by immunoblotting. Culture medium was used as blank control and HSA was used as albumin control. Results shown are representative IB (A and B) and densitometry results (C and D). n = 3 independent experiments, *P < 0.05, **P < 0.01 *vs *control.

The effect of AGE-HSA on RhoA activity and phosphorylation of ROCK was assessed by a luminescence-based G-LISA™ assay and immunoblotting. Treatment of HMVECs with AGE-HSA significantly increased RhoA activity in a time- and dose-dependent manner. Activation of RhoA reached its peak after 1 h of exposure at 50 mg/L, with the relative activity being 147.28 ± 7.6% (*P *< 0.05 vs. control) (Figure 3A and 3B). Phosphorylation of ROCK was also enhanced by AGE-HSA in a time-dependent manner and the highly phosphorylated state persisted for a long period (Figure 4A and 4B). Moreover, there was dose dependent phosphorylation of ROCK as the AGE-HSA concentration increased from 12.5 mg/L to 100 mg/L (Figure 4C and 4D). However, AGE-HSA showed no effect on ROCK protein expression, while HSA alone did not enhance activation of RhoA or phosphorylation of ROCK (Figure 3 and 4). These findings suggested that AGE-HSA enhanced the RhoA/ROCK pathway at the post-translational level.

### Suppression of RhoA with a dominant negative adenovirus prevents activation of RhoA and phosphorylation of ROCK

**Figure 5:**
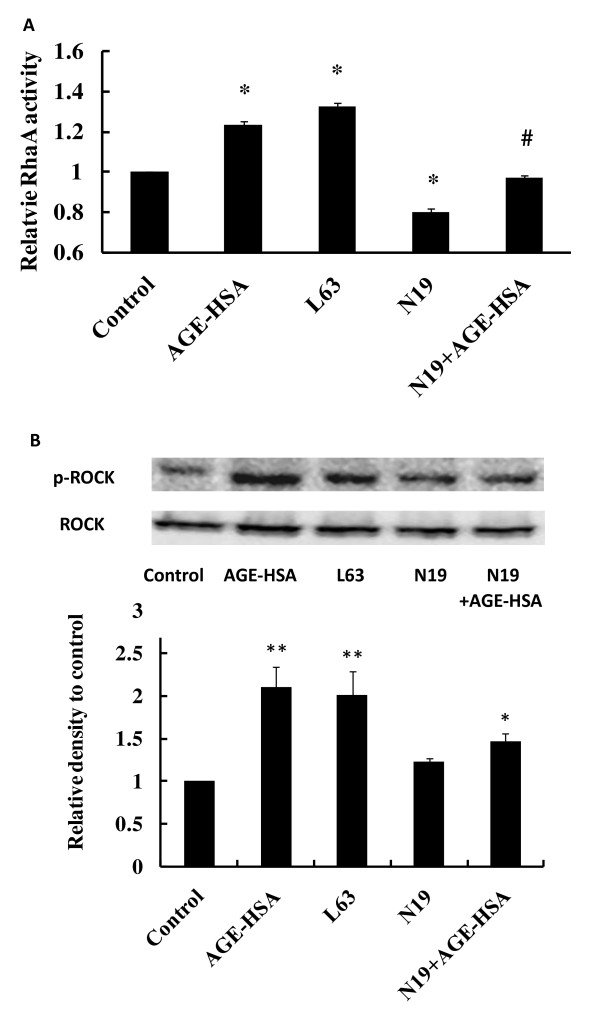
**Effect of transfection of RhoA N19 or RhoA L63 on RhoA activity and ROCK phosphorylation**. At 24 h after transfection, HMVECs were treated with 50 mg/L AGE-HSA for 60 min. RhoA activity and ROCK phosphorylation were assessed with a luminescence-based G-LISA™ assay and immunoblotting, respectively. Culture medium was used as control. *P < 0.05, **P < 0.01 *vs *control, #*P *< 0.05 *vs *AGE-HSA.

To further specify the role of RhoA/ROCK pathway in AGE-HSA-related endothelial changes, RhoA activity and ROCK phosphorylation were assessed in HMVECs treated with AGE-HSA at 24 h after infection with dominant negative RhoA N19 recombinant adenovirus. Transfection of RhoA N19 down-regulated RhoA activity in HMVECs, with relative RhoA activity being decreased from 123.56 ± 2.00% in the AGE-HSA group with the control vector to 96.86 ± 1.75% in the AGE-HSA group with RhoA N19 adenovirus (*P *< 0.05) (Figure 5A). It was also found that phosphorylation of ROCK was significantly suppressed by transfection of RhoA N19 (Figure 5B). Transfection of the constitutively activated recombinant adenovirus (RhoA L63) mimicked the effects of AGE-HSA on RhoA and ROCK in cultured HMVECs. Relative activity of RhoA increased by 32.45 ± 2.2% after active RhoA L63 transfection (*P *< 0.05) (Figure 5A), along with markedly enhanced phosphorylation of ROCK (Figure 5B).

### Down-regulation of RhoA abolishes AGE-HSA induced functional and morphological changes in HMVECs

It was indicated that down-regulation of RhoA activity by transfection of HMVECs with RhoA N19 abolished the increase of HMVEC monolayer permeability induced by AGE-HSA treatment (Figure 2B). Formation of F-actin filament bundles in AGE-HSA-treated cells was also inhibited by RhoA N19 transfection (Figure 2A), but RhoA N19 alone did not induce any changes in HMVECs. Transfection of HMVECs with constitutively activated RhoA L63 caused an increase of HMVECs monolayer permeability and disorganization of F-actin filaments. These findings confirmed the involvement of RhoA activation in endothelial barrier dysfunction caused by AGE-HSA.

### Inhibition of ROCK activation attenuates the changes of permeability and F-actin after AGE-HSA stimulation

Our previous study showed that inhibition of ROCK activation by Y-27632 could suppress the phosphorylation of moesin induced by AGE-HSA and block the AGE-evoked increase of HMVEC monolayer permeability [20]. H-1152, a more specific and stronger inhibitor of ROCK [33,34], was used in this study and its administration abolished both the formation of F-actin stress fibers and the increase of permeability stimulated by AGE-HSA (Figure 2).

### Down-regulation of RhoA activity attenuates AGE-HSA induced moesin phosphorylation

**Figure 6:**
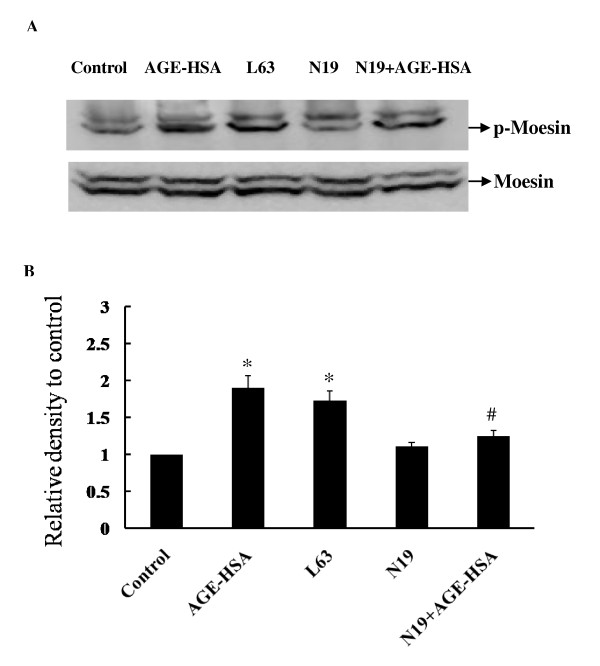
**Effect of RhoA N19 on moesin phosphorylation induced by AGE-HSA**. RhoA N19 was transfected to HMVECs 24 h before AGE-HSA (50 mg/L, 60 min) stimulation and moesin phosphorylation was detected by immunoblotting. RhoA L63 was transfected to HMVECs for 24 h as a positive control and phosphor-moesin was assayed. Culture medium was used as blank control. Results shown are representative IB (A) and densitometry results (B). n = 3 independent experiments. *P < 0.05, *vs *control, #*P *< 0.05 *vs *AGE-HSA.

To validate the effect of RhoA/ROCK pathway in AGE-HSA induced moesin phosphorylation, phospho-moesin was detected in HMVECs transfected with RhoA N19 24 h before AGE-HSA application. RhoA L63 was transfected alone to HMVECs as a positive control. The results showed that the suppression of RhoA activity by RhoA N19 obviously attenuated moesin phosphorylation induced by AGE-HSA (Figure 6A), with relative density of phospho-moesin decreasing from 1.89 ± 0.165 to 1.274 ± 0.062 (*P *< 0.05) (Figure 6B). The up-regulated activation of RhoA by RhoA L63 could mimicked the effect of AGE-HSA and significantly enhanced moesin phosphorylation (Figure 6).

### ROCK interacts directly with moesin

**Figure 7:**
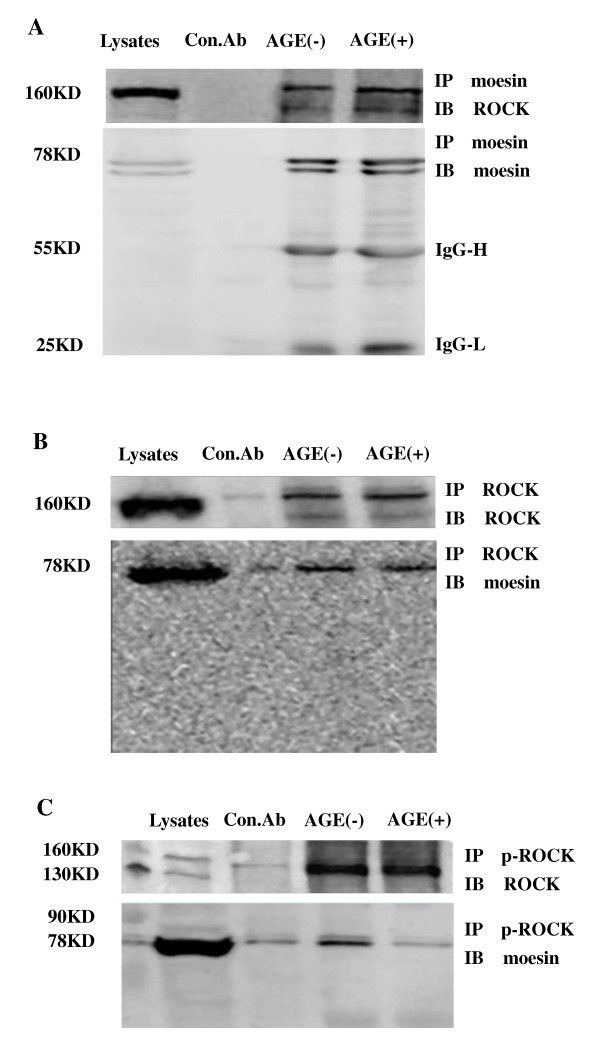
**Physical interaction between ROCK and moesin proteins is confirmed in HUVECs by immunoprecipitation (IP)- immunoblotting (IB) analysis of HUVEC whole-cell extracts after stimulation with AGE-HSA (50 mg/L,1 h)**. A: IP with moesin using mouse-antibody and IB detection with goat anti-mouse anti-ROCK antibody, ROCK was detected with or without AGE-HSA stimulation. B: IP with ROCK using rabbit antibody and IB detection with goat anti-mouse anti-moesin antibody, moesin was detected with or without AGE-HSA stimulation. C: IP with phospho-ROCK using rabbit antibody and IB detection with goat anti-mouse anti-moesin antibody, moesin was decreased after AGE-HSA stimulation. BSA was used as control albumin.

To further verify the relationship of ROCK and moesin, the direct interaction of endogenous ROCK and moesin after AGE-HSA stimulation was assessed using Immunoprecipitation (IP) - Immunoblotting (IB) analysis. HUVEC extract was obtained after stimulation with 50 mg/L AGE-HSA for 1 h. Physical interaction between ROCK and moesin was confirmed in HUVECs with or without AGE-HSA application (Figure 7A, B). The binding of moesin with phosphor-ROCK was decreased after AGE-HSA administration (Figure 7C). We speculate here that the AGE-induced phosphorylation of ROCK activated moesin and released it to act as the linker protein, resulting in the redistribution of cytoskeleton. These data demonstrated that RhoA-ROCK pathway might work through moesin activation in AGE-HSA induced endothelial responses.

### The inhibiting mutation of moesin attenuates AGE-induced phosphorylation of moesin

**Figure 8:**
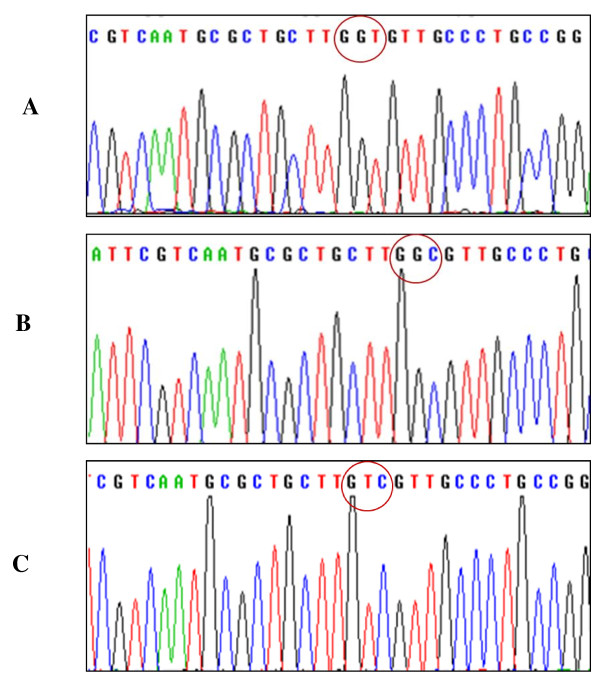
**The nucleotide sequencing of eukaryotic expressions of plasmids**. The Thr at 558 amino acid residues of moesin of pcDNA3/HA-moesin was substituted with Ala or Asp, respectively, to produce inhibited mutant pcDNA3/HA-moesin^T558A ^or activated mutant pcDNA3/HA-moesin^T558D^. The red circles marks moesin Thr 558 (A), Ala 558 (B), and Asp 558(C), respectively.

The mutant of moesin was induced by substituting threonine at 558 amino acid residues with Ala (pcDNA3/HA-moesin^T558A^) as inhibited mutant (Figure 8B), or with Asp (pcDNA3/HA-moesin^T558D^) as activated mutant (Figure 8C), respectively. The eukaryotic expressions of plasmids of two mutants and pcDNA3/HA-moesin (Figure 8A) were identified by nucleotide sequencing. The plasmids transfection efficiency was up to 30% using Lipofectamine™ LTX and PLUS™ reagents.

**Figure 9:**
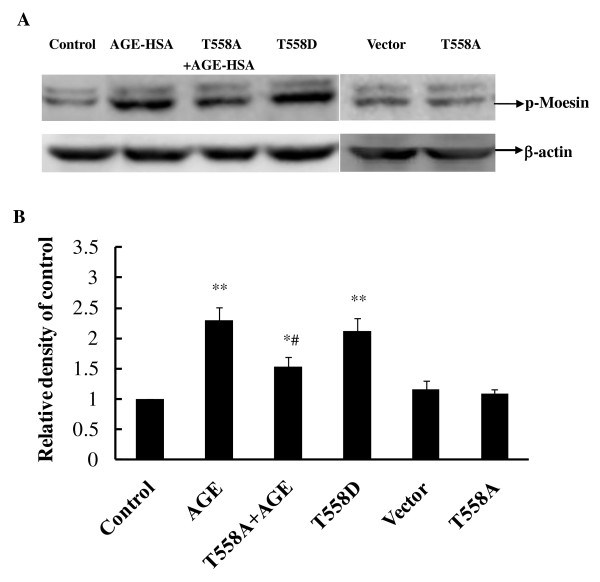
**Expression of moesin^T558A ^attenuated AGE-HSA induced phosphorylation of moesin**. HUVECs transfected with plasmid pcDNA3/HA-moesin^T558A ^were stimulated with 50 mg/L AGE-HSA for 1 h. Plasmid pcDNA3/HA-moesin^T558D ^was transfected to HUVECs without AGE-HSA stimulation. The phosphorylation of moesin was detected with immunoblotting. Vector pcDNA3/HA or inhibited moesin mutant pcDNA3/HA-moesin^T558A ^alone was also transfected to HUVECs respectively. Results shown are representative IB (A) and densitometry results (B). n = 3 independent experiments. * *P *< 0.05 and ** *P *< 0.01 *vs *control, # *P *< 0.05 *vs *AGE-HSA.

The transfection of plasmid pcDNA3/HA-moesin^T558A ^into HUVECs decreased the level of phosphorylated moesin after 1 h of exposure to AGE-HSA at 50 mg/L, while the transfection of plasmid pcDNA3/HA-moesin^T558D ^itself could educe phosphorylation of moesin without AGE-HSA application (Figure 9). The transfection of control vector pcDNA3/HA and inhibited moesin mutant pcDNA3/HA-moesin^T558A ^alone into HUVECs exerted no effect on level of moesin phosphorylation. These results directly indicated that AGE-HSA caused the phosphorylation of threonine in 558 residues of moesin.

### The inhibiting mutation of moesin attenuates AGE-induced endothelial hyper-permeability and F-actin disorganization

**Figure 10:**
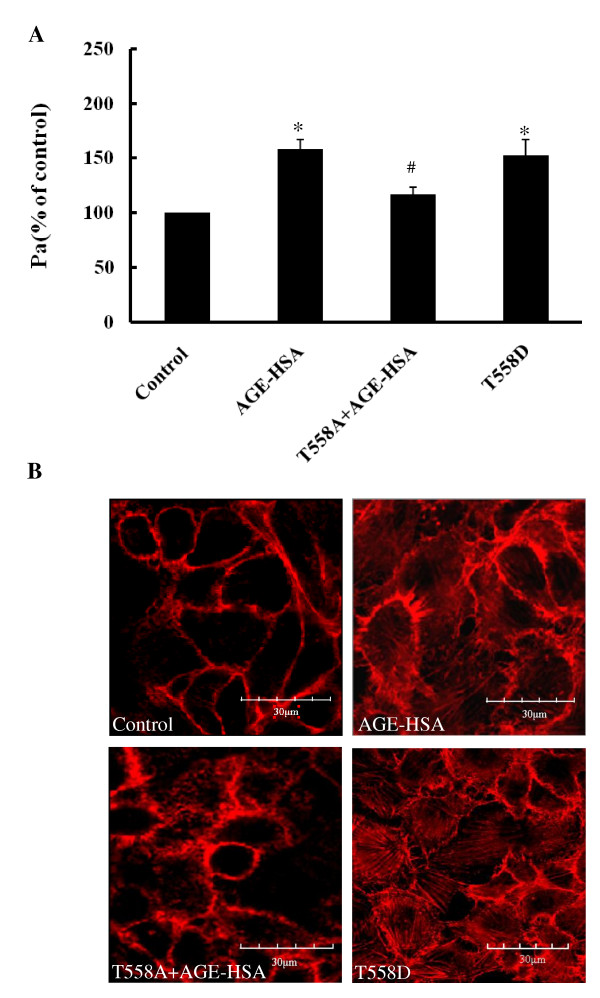
**Expression moesin^T558A ^decreased the hyper-permeability response (A, n = 3) and F-actin redistribution (B) after AGE-HSA stimulation**. HUVECs were stimulated 1 h by 50 mg/L AGE-HSA after transfected with plasmid pcDNA3/HA-moesin^T558A ^for 24 h, or transfected with plasmid pcDNA3/HA-moesin^T558D ^without AGE-HSA stimulation. Representative confocal images of F-actin organization and the formation of stress fiber in HUVECs were showed with similar results observed in 3 different cultures. * *P *< 0.05 vs control, # *P *< 0.05 vs AGE-HSA.

Our previous reports have shown that AGE-HSA increased the monolayer permeability and caused the formation of F-actin stress fiber in HUVECs and HMVECs [15,20]. In this present study, the transfection of plasmid pcDNA3/HA-moesin^T558A ^into HUVECs decreased the hyper-permeability response induced by AGE-HSA administration (50 mg/L, 1 h) from 158.34 ± 4.17% to 116.67 ± 3.21%. It also preserved the distribution of F-actin in endothelial cortex area after AGE-HSA stimulation. The transfection of plasmid pcDNA3/HA-moesin^T558D ^itself increased endothelial monolayer permeability to 152.62 ± 7.14%, also triggered the formation of F-actin stress fiber (Figure 10). These results provide direct evidence for the involvement of phosphorylation of moesin in Thr 558 residues in AGE-induced endothelial dysfunction.

## Discussion

It is well known that Rho GTPases play a central role in the functional regulation of EC barrier function [35]. RhoA/ROCK-dependent modulation of actin cytoskeleton organization and cell contractility are involved in the regulation of endothelial permeability [18,36,37]. We have previously demonstrated that ROCK was phosphorylated by AGE stimulation and that suppression of ROCK activity by the inhibitor Y-27632 attenuated the AGE-dependent increase of permeability in ECs [20]. In the present study, we demonstrated the involvement of the RhoA/ROCK pathway in cellular responses to AGEs by showing time- and dose-dependent increases of RhoA activation and ROCK phosphorylation in HMVECs exposed to AGE-HSA. Then, using a recombinant adenovirus containing dominant negative human RhoA (RhoA N19), we showed that the RhoA activity of transfected HMVECs was significantly down-regulated and these cells showed much weaker responses to AGE-HSA stimulation in terms of RhoA activation and ROCK phosphorylation compared with naive HMVECs. Transfection of this virus also attenuated changes of the F-actin cytoskeleton and monolayer permeability induced by AGE-HSA. On the other hand, infection with recombinant adenovirus containing constitutively activated human RhoA (RhoA L63) led to increased RhoA activation and ROCK phosphorylation, as well as rearrangement of F-actin in HMVECs and increased permeability of HMVEC monolayers. H-1152, a specific ROCK inhibitor, abolished the AGE-induced disarrangement of F-actin and endothelial barrier disruption. These findings is the first to demonstrate the specific effects of RhoA activation by actually showing the alteration of RhoA activity and the subsequence of down-regulating RhoA expression in AGE-induced endothelial dysfunction. These data, together with our previous results [20], emphasizes the role of the RhoA/ROCK pathway in AGE-induced endothelial dysfunction.

Hirose et al. have reported that by promoting spontaneous formation of the RhoA and RAGE complex, AGE-BSA induces reorganization of the actin cytoskeleton through RAGE/RhoA activation, leading to increased permeability of HUVECs [14]. Our previous study also revealed a role of RAGE in AGE-induced phosphorylation of ROCK [20]. This present study used HMVECs, which are the cells involved in controlling vascular barrier function, to verify the influence of AGEs on vascular endothelial barrier function. Using dominant negative RhoA N19 and constitutively activated RhoA L63, this study is the first to specifically demonstrate the involvement of the RhoA-ROCK pathway in AGE-related cellular responses.

Various signaling mechanisms, including NAD(P)H and reactive oxygen species, p38 MAPK, phospholipase C, and calmodulin-dependent protein kinase [2,20,32,38], have been suggested to have a role in AGE-induced cellular dysfunction. It would be interesting to also assess the relation of Rho/ROCK to other pathways involved in AGE-induced endothelial responses. We found that inhibition of ROCK with H-1152 attenuated AGE-HSA-induced phosphorylation of p38 in HMVECs. Interestingly, inhibition of p38 activation with SB203580 also suppressed the phosphorylation of ROCK (data not shown). While there is considerable evidence that RhoA/ROCK regulates p38 MAPK activation [39], p38 might also regulate RhoA/ROCK activity [40], i.e., RhoA or p38 could be upstream regulators of each other [39,41,42]. However, more research is needed to clarify their complicated relation in the responses to AGE-HSA.

It is necessary to extend the downstream target of Rho/ROCK since ROCK did not directly interacts with F-actin. It has been well documented that activation of RhoA/ROCK can phosphorylate and inactivate myosin light chain phosphatase (MLCP), hence promoting MLC phosphorylation and acto-myosin contraction in endothelial cells [43-47]. Another protein family, ERM protein family, has also emerged as the possible candidate target for ROCK in regulation of endothelial response [21-23]. There are three members, ezrin, radixin, and moesin in ERM protein family. A new report has indicated the other members of ERM family, ezrin and radixin, might also play a role in 2-methoxyestradiol induced modulation of permeability human pulmonary artery endothelial cells (HPAEC) [48]. But the expression and phosphorylation of ERM can vary from endothelium to endothelium and/or from stimuli to stimuli. In our previous report [20], we detected the total ERM protein expression and found out that treatment with moesin siRNA not only efficiently inhibited the protein expression of moesin, but also total ERM in HMVECs. While moesin expression was almost totally depressed by siRNA, total ERM expression was almost totally depressed either. This result indicated that in HMVECs, moesin is the major ERM protein. Functionally, down-regulation of moesin expression by siRNA prevented AGE-induced cytoskeletal changes and permeability increases in HMVECs. These results convinced us that moesin is the major ERM in regulation of endothelial function, especially in HMVECs. Of course, we could not rule out the involvement of other members of ERM family, ezrin and radixin, in regulation of endothelial function since we did not monitor the phosphorylation of ezrin and radixin in this study. Even the antibody is moesin specific, according to the manufacture datasheet, there is still a possibility that the upper band in Figure 6 probably indicated ezrin or radixin too, since the antibody used here can partially detects ezrin phosphorylated at Thr 567 and radixin phosphorylated at Thr 564.

Previously, we have demonstrated that the inhibition of ROCK with Y-27632 attenuated the AGE-induced phosphorylation of moesin in HMVECs [20]. The data in this present study indicated again that moesin is the downstream target of RhoA/ROCK pathway in AGE-induced endothelial response. The AGE-HSA induced moesin phosphorylation in Thr558 residue was remarkably attenuated by down-regulation of RhoA activation with dominative negative recombinant adenovirus RhoA N19. The direct activation of RhoA by constitutively active recombinant adenovirus RhoA L63 enhanced the phosphorylation of moesin (Figure 6). This study further provides a direct evidence to confirm the physical interaction of ROCK and moesin by using IP-IB analysis. Moesin was immunoprecipitated with ROCK in unstimulated cell lysates and AGE stimulation did not alter the binding of ROCK and moesin (Figure 7A, B). It is consistent with Hébert et al's report that ezrin and moesin are immunoprecipitated with ROCK without stimulation in Jurkat cells [49], indicating that ROCK can bind moesin. However, the binding of phosphor-ROCK with moesin decreased significantly after AGE application (Figure 7C). We speculate here that the activated ROCK triggered the phosphorylation of moesin and released moesin from this binding complex to act as linking protein, resulting in the reorganization of F-actin.

It has been revealed that tyrosine and threonine residues are major phosphorylation sites in ERM protein [23,50,51]. By using specific antibody against phosphor-Thr moesin, Koss et al. has demonstrated that vascular permeability increasing mediator, such as TNF-α, could phosphorylate threonine 558 of moesin and enhance the hyper-permeability response in pulmonary microvascular endothelial cells [52]. In this present study, while the inhibited mutant plasmid pcDNA3/HA-moesin^T558A ^was introduced to HUVECs, the AGE-induced phosphorylation of moesin was attenuated (Figure 9), as well as the F-actin disorganization and endothelial barrier dysfunction (Figure 10). The activated mutant plasmid pcDNA3/HA-moesin^T558D ^mimicked the AGE-evoked endothelial response with formation of F-actin stress fiber and increase of endothelial monolayer permeability. These data are consistent with the results from Zhou et al, indicating that exogenously expressed meosin bears biochemical characteristics similar to endogenous protein [53]. Although it is yet to rule out the possibility of AGE-induced tyrosine phosphorylation since the state of phosphotyrosine was not detected in this study, the results have demonstrated that threonine 558 is the phosphorylation site of moesin in AGE-stimulated endothelial cells.

Taking together, this study provides direct evidences to show that the up-regulation of RhoA activity is involved in AGE-induced endothelial dysfunction. ROCK physically interacts with moesin and the RhoA-triggered phosphorylation of ROCK results in phosphorylation of threonine at 558 residue in moesin. This RhoA/ROCK -dependent moesin phosphorylation regulates AGE-induced endothelial dysfunction.

## Abbreviations

AGE: Advanced glycation end products; AGE-HSA: AGE-modified human serum albumin; DMSO: Dimethylsulfoxide; EC: Endothelial cell; ERM: Ezrin/radixin/moesin; FBS: Fetal bovine serum; HMVEC: Human dermal microvascular endothelial cell; HSA: Human serum albumin; HUVECs: Human umbilical vein endothelial cells; MAPK: Mitogen activated protein kinase; PBS: Phosphate buffered saline; PCR: Polymerase chain reaction; QPCR: Real-time quantitative PCR; RAGE: Receptor of AGE; Rho: Ras homologue oncogene; RNA: ribonucleic acid; ROCK: Rho kinase; RT-PCR: reverse transcription polymerase chain reaction.

## Competing interests

The authors declare that they have no competing interests.

## Authors' contributions

QH conceived the study, arranged the collaboration, initiated the manuscript, edited and compiled the final version for submission. JW and HL participated in its design and coordination. JW, HL, BCh, QL, XH, LW, XG performed Laboratory analyses and study design. All authors read and approved the final manuscript.
